# Multi-Scale Extension in an Entorhinal-Hippocampal Model for Cognitive Map Building

**DOI:** 10.3389/fnbot.2020.592057

**Published:** 2021-01-14

**Authors:** Jiru Wang, Rui Yan, Huajin Tang

**Affiliations:** ^1^College of Computer Science, Sichuan University, Chengdu, China; ^2^College of Computer Science and Technology, Zhejiang University of Technology, Hangzhou, China; ^3^College of Computer Science and Technology, Zhejiang University, Hangzhou, China

**Keywords:** path integration, place cell, grid cell, spatial cognition, cognitive map building

## Abstract

Neuroscience research shows that, by relying on internal spatial representations provided by the hippocampus and entorhinal cortex, mammals are able to build topological maps of environments and navigate. Taking inspiration from mammals' spatial cognition mechanism, entorhinal-hippocampal cognitive systems have been proposed for robots to build cognitive maps. However, path integration and vision processing are time-consuming, and the existing model of grid cells is hard to achieve in terms of adaptive multi-scale extension for different environments, resulting in the lack of viability for real environments. In this work, an optimized dynamical model of grid cells is built for path integration in which recurrent weight connections between grid cells are parameterized in a more optimized way and the non-linearity of sigmoidal neural transfer function is utilized to enhance grid cell activity packets. Grid firing patterns with specific spatial scales can thus be accurately achieved for the multi-scale extension of grid cells. In addition, a hierarchical vision processing mechanism is proposed for speeding up loop closure detection. Experiment results on the robotic platform demonstrate that our proposed entorhinal-hippocampal model can successfully build cognitive maps, reflecting the robot's spatial experience and environmental topological structures.

## 1. Introduction

Based on the inner spatial representation mechanism in the brain, animals can do navigation and complex high-level cognitive tasks. Spatial cells with regular spatial firing patterns in the brain are considered to be related (Moser et al., 2008), and these mainly include head direction cells (Taube, 2007), grid cells (Fyhn et al., 2004b), and place cells (O'Keefe and Dostrovsky, 1971). Deciphered by neuroscience studies, cognitive map theory opened up a new paradigm for modeling spatial cognition, which provides great insight into bio-inspired mapping and navigation (Milford and Wyeth, 2010; Yuan et al., 2015; Tang et al., 2016). The nature of the cognitive map mechanism that supports space-related tasks, however, remains vague.

The discovery of place cells (O'Keefe and Dostrovsky, 1971) marks the beginning of a new era where scientists in cognitive map fields started to explore the spatial perception and navigation functions of mammals at the neuron level. Place cells are mainly located in the CA1CA3 regions of the hippocampus. A specific place cell fires when an animal is at a certain position (the place field of the cell), and different place cells fire at different locations. The entire environment can be represented by place cells' activities throughout the hippocampus, and they are therefore considered to serve as a basic component of spatial representation. Grid cells are discovered in the medial entorhinal cortex (MEC), which is closely related to the hippocampus. They show sharply tuned place cell-like spatial firing patterns, but each has multiple firing fields (Fyhn et al., 2004b). These fields formed hexagonal grids, tiling the entire environment. Furthermore, it has been demonstrated that multiple spatial scales in grid cells increase in discrete steps along the dorsoventral axis, and each successive scale is suggested to increase by a fixed factor (Barry et al., 2007; Stensola et al., 2012). Defined by three parameters: spatial scale (distance between firing fields), orientation (direction relative to an external reference axis), and phase (displacement relative to an external reference point), grid cells are indicated to be a key component of path integration in rodents' spatial cognition (Hafting et al., 2005; Moser et al., 2014; Banino et al., 2018). Head direction cells were discovered in neighboring regions of the hippocampus and are characterized by responding to an animal's head direction. One single cell fires when the animal's head is at a specific orientation and is not influenced by location (Taube, 2007). Head direction cells can provide directional information like a compass during the animal's exploration in environments. Cognitive map theory generally posited that head direction cells provide direction information, grid cells perform path integration, and place cells are responsible for place representation.

When rats explore unfamiliar environments, they can keep track of relative displacement and rightly return to the original locations depending on inner path integration, including angular integration in head direction system and path integration in grid cell system (McNaughton et al., 2006). Head direction cells can be modeled through integrating angular velocity into a one-dimensional continuous attractor network (CAN). In general, computational models describing grid cell firing pattern formation are divided into two classes: oscillatory interference models (Burgess, 2008; Zilli and Hasselmo, 2010) and CAN models (Fuhs and Touretzky, 2006; Burak and Fiete, 2009). Given the former faces limitations as a candidate mechanism for spatial periodicity (Burak and Fiete, 2009; Moser and Moser, 2013), more and more researchers have become interested in CAN. By integrating linear and angular velocity (Fyhn et al., 2004b; McNaughton et al., 2006; Burak and Fiete, 2009), path integration in two dimensions can be modeled by a two-dimensional CAN. Grid cells in the CAN are arranged in a 2D sheet (layer or module), and a recurrent connection among cells in the same layer with a global inhibition makes the random patterns of population activity spontaneously merge into organized “bumps” of grid cell population activity (Burak and Fiete, 2009). The bumps are envisaged to move as the animal moves from one place to another. The firing pattern of a single grid cell is obtained by averaging the accumulated firing rates over the whole exploration of environments. Hafting et al. first showed that grid cells in MEC possibly support a two-dimensional continuous attractor-based representation of the environment (Hafting et al., 2005). O'Keefe et al. further described more details of modeling grid cells (O'Keefe and Burgess, 2005). Fuhs et al. first implemented a path integration model based on attractor dynamics and periodic spatial firing fields with regular hexagonal grid patterns are created (Fuhs and Touretzky, 2006). Moser et al. also maintained a more positive and optimistic attitude to the CAN (Moser and Moser, 2013; Moser et al., 2014). Despite requiring further investigation, they suggest that most of the available evidence supports the attractor theory. This suggestion is also shared by other recent studies (Burak and Fiete, 2009; Giocomo et al., 2011; Zilli, 2012; Schmidt-Hieber and Hausser, 2014). In addition, there are works, from the deep learning aspect, trying to support neuroscientific theories about the critical role grid cells play in spatial cognition. They trained recurrent networks to perform path integration, leading to the emergence of a neural response resembling firing patterns in grid cells (Banino et al., 2018; Cueva and Wei, 2018). Place cells are generally considered to be driven by grid cells' activities, and feedforward networks can be used to form place cell activities. Weighted connections between them can be established by unsupervised learning (Fuhs et al., 2005; McNaughton et al., 2006; Solstad et al., 2006), such as Hebbian learning or competitive learning (Rolls et al., 2007; Savelli and Knierim, 2010; Monaco and Abbott, 2011; de Almeida et al., 2012; Zeng and Si, 2017). The appropriate combination of grid cells across layers can achieve a unique identification for the current environmental location, and these are represented by place cells (Hartley et al., 2014).

By modeling spatial cells and integrating their activities, the neural circuitry in the brain related to spatial cognition can be simulated to do dead reckoning but with error accumulation. With the assistance of external sensory information, effective cognitive map building of the real environment can be achieved and applied to a mobile robot; this is called the bio-inspired Simultaneous Localization And Mapping (SLAM) system. Cuperlier et al. built a neurobiologically inspired mobile robot navigation system using a new cell type which they named transition cell, which represents both position and direction of movement or spatiotemporal transitions (Cuperlier et al., 2007). Significant progress was made by Milford and Wyeth (2008) in emulating the spatial navigation ability of the brain, which can build a semi-metric topological map in a real and large area. This work has been extended to use an RGB-D sensor to build environmental cognitive maps for robot navigation (Tian et al., 2013; Shim et al., 2014). Jauffret et al. used a mathematical model of grid cells for a mobile robot navigation system in which adding visual input to recalibrate path integration fixes noisy path integration input, thus sharpening grid cells' firing patterns (Jauffret et al., 2015). Yoan et al. proposed a neural architecture to tackle the localization challenge for autonomous vehicles based on a neurobotic model of the place cells found in the hippocampus of mammals (Espada et al., 2018, 2019).

In this paper, an enhanced cognitive map building system is designed for a mobile robot in real environments in which we propose an optimized path integration mechanism of grid cells and novel hierarchical vision processing methods to achieve a workable map building system. Here, the path integration is completed through grid cells with different scales, which can be obtained by multi-scale extension of a basic single-scale model. We present an optimized grid cell modeling mechanism that enhances the model's ease of use in a multi-scale extension. In addition, visual processing is also a key part that cannot be ignored when it comes to time performance improvement. A novel visual template organization method based on a hierarchical structure was proposed to speed up scene matching and loop closure detection. Moreover, weighted connections from grid cells to place cells are established by simple and off-line unsupervised learning, which is a common solution for spatial representation and not environment-specific. Combining real-time path integration of grid cells, stable place representation of place cells, and the vision-assisted map correction mechanism, the successful transition from a computational model to robotic application in real environments is achieved. Meanwhile, as an extension of this work, we demonstrate the potential coding advantage of grid cells in motion planning by achieving grid cell-based multi-scale motion planning.

## 2. Materials and Methods

### 2.1. The System Architecture

**Figure 1:**
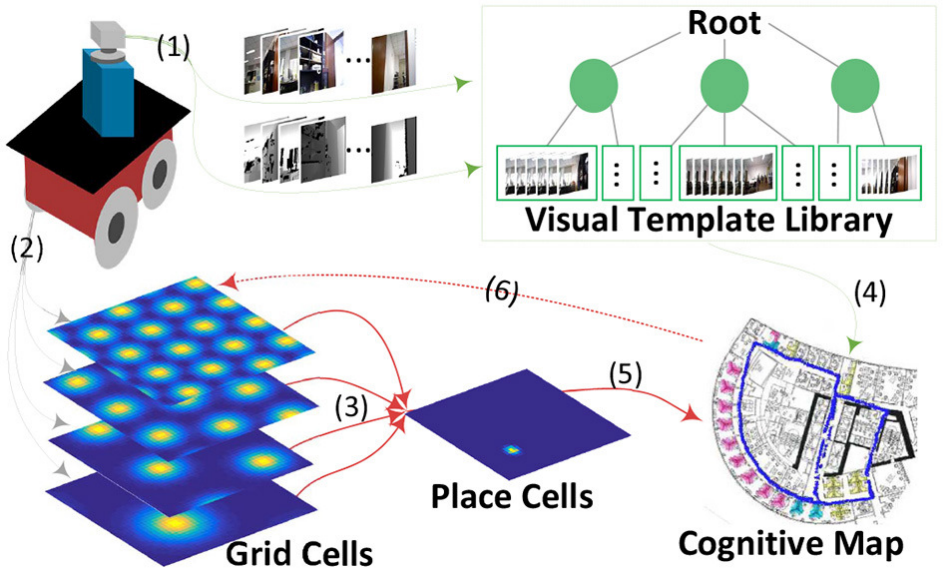
A cognitive map-building architecture based on a mobile robot platform in real environments. The velocity input signals derived from the robot's wheel encoders drive the grid cell model for path integration (2), which is projected to place cells for spatial location decoding (3&5). The vision process module obtains RGB and depth information of the environment from the robot's RGB-D sensor (1). This information is organized into a visual template library based on tree structure for loop closure detection(4) after which the cognitive map is corrected and neuronal activities are reset (6).

Figure 1 shows the system architecture, a cognitive map building model based on a mobile robot platform in real environments. The whole framework includes six major components:

1. Capturing vision information, including RGB and depth data provided by an RGB-D sensor mounted on the front top of the mobile base.
2. Multi-scale path integration with self-motion information as the driving force, including angular and linear velocity obtained from wheel encoders equipped on the front wheels of the robot mobile base.
3. Stable place encoding with learned weighted connections from the multi-scale path integration part.
4. A novel hierarchical VTT (Visual Template Tree) for visual template organization, speeding up visual scene matching and further facilitating the time performance of the system.
5. A topological map reflecting the current environment; it is built and updated during the robot's environmental exploration.
6. Accumulated error correction in path integration; it is followed by grid cell activity resetting when loop closures are detected.

### 2.2. Multi-Scale Grid Coding for Path Integration

Although the exact mechanism of hexagonal firing patterns in grid cells remains unknown, signals about velocity and direction can be integrated by interconnected cells in the CAN. Since the population of grid cells in one CAN have the same spatial scale, then multiple populations of grid cells corresponding to multiple CANs can achieve multi-scale extension and path integration. In this section, a modified dynamical model for grid coding and optimized weight distribution are given for achieving a more easy-to-use path integration mechanism of multi-scale grid cells.

#### 2.2.1. The Modified Grid Coding Dynamics

For CAN-based grid cell coding, a basic model is considered to complete multi-scale extension, which must be highly extensible and easy to use. A representative example is the single-layer (sheet or module) CAN proposed in Burak and Fiete (2009) (hereinafter, Burak's model). Yuan et al. used this basic model for completing an 80-layer model(hereinafter, Yuan's model) (Yuan et al., 2015), each layer with different spatial scales. The dynamics of the basic model is as follows:

(1) $$\begin{array}{l} \tau dg_{i}/dt=-g_{i}+s\left(\underset{j}{\sum }W_{ij}g_{j}+I_{i}\right) \end{array}$$

(2) $$\begin{array}{l} I_{i}=1+\alpha {\hat{\text{e}}}_{\theta_{i}}.{\text{v}}_{t} \end{array}$$

where *s*(*x*) = 0 if *x* ≤ 0; otherwise, *s*(*x*) = *x*. *g*_*j*_ is the activity of neuron *j* and τ the time-constant of neuron response. There are *N* × *N* neurons in one neural sheet, and each neuron *i* has a preferred direction θ_*i*_. The feed-forward excitatory input to neuron *i* is defined by *I*_*i*_, where α is the gain factor of the velocity response of the network, ${\hat{\text{e}}}_{\theta_{i}}$ is the unit vector pointing to θ_*i*_, and **v**_*t*_ is the velocity at time *t*. All grid cells have random initial firing rates and the driving velocity is initially set to 0 during some time for forming stable grid firing patterns. To adapt grid cells to the needs of multi-scale extension, the above dynamical model is modified and the sigmoidal function is utilized as the activation function. The modified dynamics are as follows:

(3) $$\begin{array}{l} \tau dg_{i}/dt=-g_{i}+s\left(\underset{j}{\sum }W_{ij}g_{j}+I_{i}\right) \end{array}$$

(4) $$\begin{array}{l} s\left(x\right)=1/\left(1+exp\left(-k_{g}x\right)\right) \end{array}$$

(5) $$\begin{array}{l} I_{i}=\alpha {\hat{\text{e}}}_{\theta_{i}}.{\text{v}}_{t} \end{array}$$

where *k*_*g*_ represents the steepness of the sigmoidal curve. The sigmoidal function is a bounded non-linear transfer function that can limit input from other neurons and external environments within a certain range.

**Figure 2:**
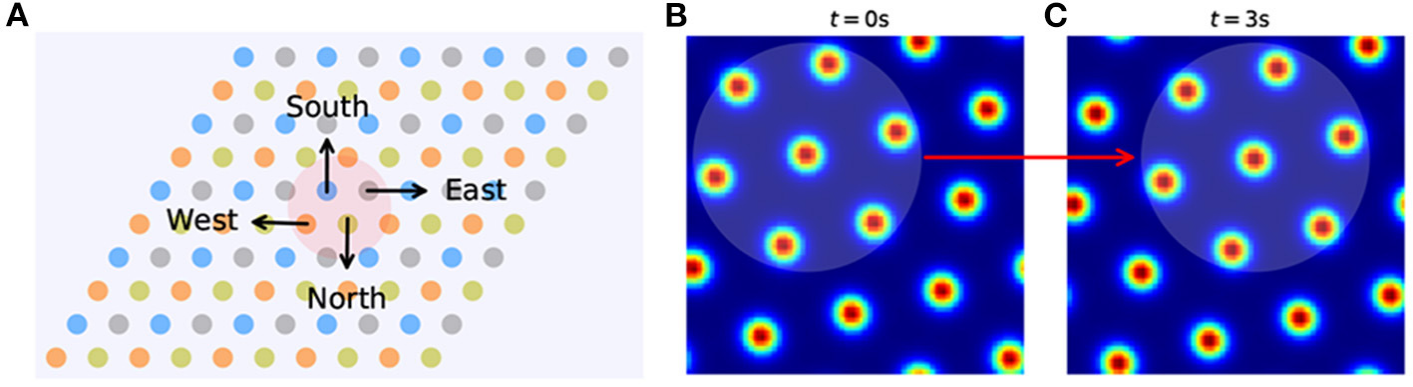
The grid neural sheet. **(A)**, Grid cells are arranged in a 2D neural sheet, each with a preferred direction (West, East, North, and South). The sheet is subdivided into many sub-units (right) and each sub-unit contains four grid cells with all preferred directions. **(B,C)** The activity bumps move in the grid neural sheet from *t* = 0 to *t* = 3 s.

By analyzing the activity change in grid cells, we can interpret how input about running velocity drives the path integration process. The preferred direction of a grid cell is related to the velocity input it receives. For rats, preferred directions might show continuous variation within the range of 0 to 2π. Here, for convenience in grid cell modeling, they are restricted to North, East, South, and West, and these are represented by π/2, 0, 3π/2, and π, respectively. The grid neural sheet can be considered as including (*N* × *N*)/4 sub-units, each consisting of 2 × 2 neurons (Figure 2A). The preferred directions of every four grid cells in one unit are denoted as ***θ***. If the velocity input is ${\text{v}}_{t}={\left[v_{x}\;v_{y}\right]}^{T}$ at time *t*, the feed-forward excitatory input ***I*** into four neurons in one unit are as shown below:

$$\begin{array}{ll} \theta =\left[\begin{array}{cc} \theta_{1} & \theta_{2}\\ \theta_{3} & \theta_{4} \end{array}\right]=\left[\begin{array}{cc} \pi /2 & 0\\ \pi & 3\pi /2 \end{array}\right]\Rightarrow I & =\left[\begin{array}{cc} I_{1} & I_{2}\\ I_{3} & I_{4} \end{array}\right]=\left[\begin{array}{cc} \alpha v_{y} & \alpha v_{x}\\ -\alpha v_{x} & -\alpha v_{y} \end{array}\right] \end{array}$$

Then the activity of neurons will increase or decrease with the change of velocity input and result in the movement of activity bumps in the grid neural sheet (Figures 2B,C), which is consistent with the movement of the rat. By continuing this process, the path integration in space can be achieved by the smooth movement of activity bumps of the grid cell population in the neural sheet.

#### 2.2.2. The General Multi-Scale Extension Mechanism

The neuroscientific discovery about discrete grid scales (Barry et al., 2007; Stensola et al., 2012) provides us with theoretical guidance to optimize Yuan's model. Experimental recordings show that grid cells are organized into discrete modules with the scale increasing in discrete steps. There are only four or five levels of the grid scale, and each level is referred to as a module. The ratio between spatial scales of each successive pair of modules is between 1.4 and 1.7. As shown in CAN-based grid cell modeling, all cells in one sub-CAN have the same spatial scale and direction and different phases. Different grid cells will fire together at the same spatial location, and different sets of grid cells will fire at different locations. Encoding spatial information through the combinatorial coding of grid cells, despite only being several discrete scales, can still provide an efficient way of representing environmental positions. This organization mechanism of the grid scale may achieve space encoding with a minimum number of grid cells. Burak's and Yuan's models complete the single-scale and multi-scale grid cell modeling, respectively, based on CAN. However, neither gave instructive conclusions and guidelines about multi-scale extension when grid cells are used for path integration. In Yuan's model, spatial scales of grid cells increase in a step-like manner, and then the corresponding grid period parameter λ increases linearly ranging from 13 to 21. Then 80 grid cell modules for path integration are generated, leading to dramatic increase of system computational effort. Moreover, it is observed that firing patterns in some grid modules were unsatisfactory and unqualified to be a hexagonal grid.

**Figure 3:**
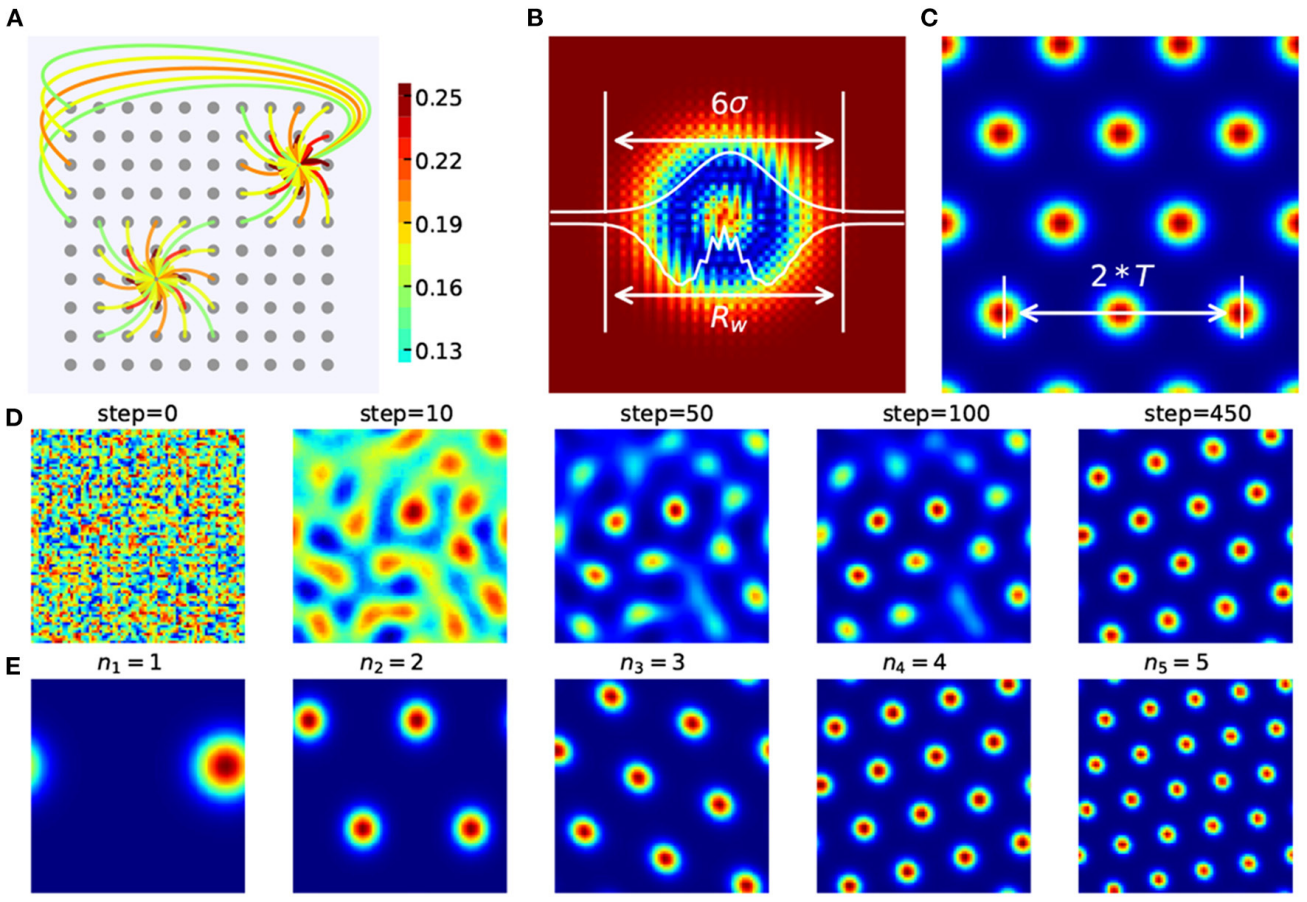
Recurrent weight connections in CAN-based grid cell modeling. **(A)** The periodic and recurrent connections between grid cells. **(B)** The heat map and one-dimensional profiles of *W*_*ij*_ between grid cells. According to the 3σ rule in probability distribution, *R*_*w*_ can be approximated by six times the standard deviation σ in *W*_*ij*_. **(C)** *R*_*w*_ is roughly twice the grid cell's period *T*. **(D)** A stable spatially firing pattern with specific scale is gradually formed. **(E)** Spatially periodic firing patterns of grid cells with different scales are generated in our grid cell model in which *n*_*m*_ represents the number of periods.

The distance-related recurrent connections (Figure 3A) between grid cells are the key to form spatially periodic bumps and also the key to multi-scale extension. In the basic model they used, *W*_*ij*_ is the synaptic weight from neuron *j* to neuron *i* and can be demonstrated:

(6) $$\begin{array}{l} W_{ij}=a*exp\left(-\gamma |\text{x}{|}^{2}\right)-exp\left(-\beta |\text{x}{|}^{2}\right) \end{array}$$

with

(7) $$\begin{array}{l} \text{x}={\text{x}}_{i}-{\text{x}}_{j}-l{\hat{\text{e}}}_{\theta_{j}} \end{array}$$

where *a* is a constant and *l* is the shift in the outgoing weights. In Burak's simulations, *a* = 1, γ = 1.05β, β = 3/λ^2^, and λ is approximately the periodicity of the formed lattice in the grid sheet.

In the above, the spatial scale of grid cell is determined by the weight matrix *W*_0_ (depending on γ, β, and λ) and the gain of the network's flow response to a velocity input (depending on *l* and α). Simply tuning λ fails to make grid cells achieve spatial scales we need. When it comes to multi-scale extension, more than one parameter should be taken into account to accommodate different network sizes and grid scale, which brings inconvenience. The activity level in networks due to both the size and number of activity packets could be controlled by the level of lateral inhibition between neurons. In this paper, the recurrent weight setting is modified and defined as follows:

(8) $$\begin{array}{l} W_{ij}=a_{m}\left(exp\left(-\frac{b|\text{x}{|}^{2}}{2\sigma_{m}^{2}}\right)-exp\left(-\frac{|\text{x}{|}^{2}}{2\sigma_{m}^{2}}\right)\right) \end{array}$$

(9) $$\begin{array}{l} a_{m}=\frac{w_{0}*\sqrt{n_{m}}}{max\left(\sqrt{n}\right)} \end{array}$$

where *b* = 1.01 is the same for all *M* grid modules and ***n*** = (*n*_1_, *n*_2_, ⋯ , *n*_*M*_) represents the number of periods of grid cells' firing patterns. σ_*m*_ denotes the gaussian parts' standard derivation in the *m*th grid module's weight matrix. As shown in Figure 3D, a stable spatial firing pattern of grid cell with 4 periods is gradually formed.

**Figure 4:**
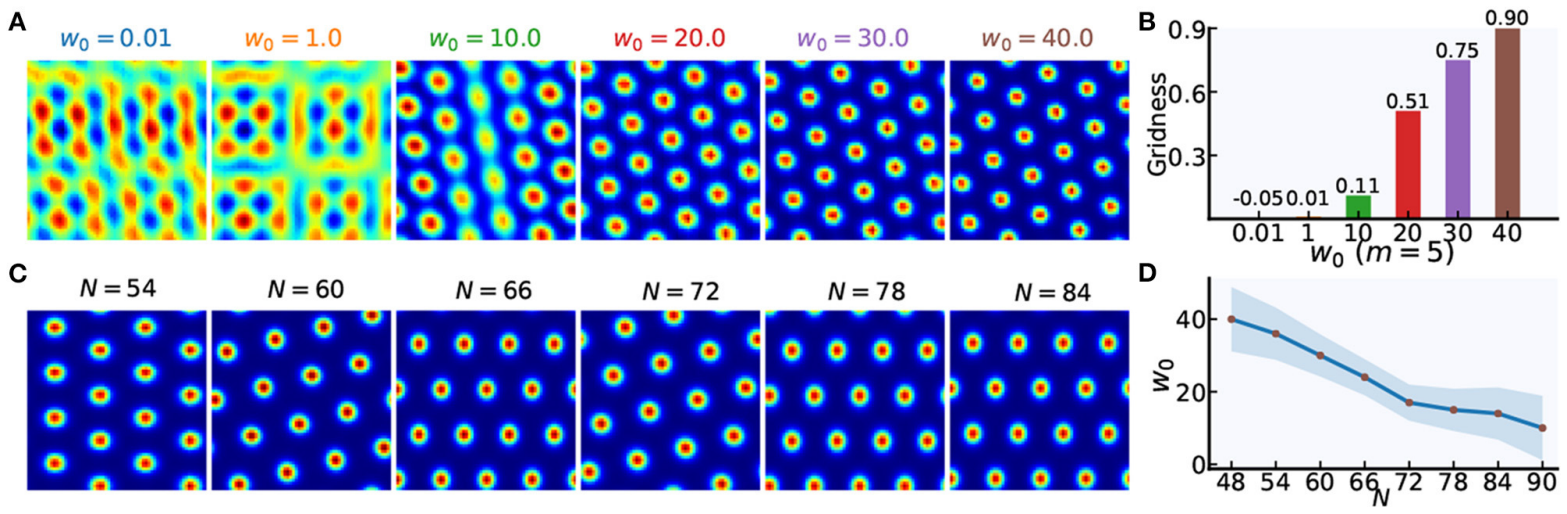
The weight gain setting in weighted connections between grid cells. **(A)** Taking *m* = 5 as an example, spatially periodic bumps are gradually formed with the increase of the weight gain factor *w*_0_. **(B)** The gridness scores corresponding to firing patterns in **(A)**. **(C)** Taking *m* = 4 as an example, spatially periodic firing patterns can be achieved in grid cell models with different network size (*N* × *N*). **(D)** With the increase in network size (*N* × *N*), the weight gain factor *w*_0_ will be lowered to achieve high-quality hexagonal firing patterns of grid cells.

The weight gain factor *w*_0_ modulates the recurrent connection strength between grid cells. Stronger recurrent connections can helpful for forming periodically organized bumps in neural sheets, as shown in Figures 4A,B. Taking the grid cell model with *N* = 48 as an example, spatially periodic bumps gradually emerge in the neural sheet with an increase of *w*_0_. Furthermore, the larger value of *N*, the smaller value of *w*_0_ for forming grid firing patterns of high quality. Figure 4D show us that the relationship of *w*_0_ and *N*. When the network size of each grid module increases from 48 × 48 to 84 × 84, the suitable value of *w*_0_ will gradually decrease.

σ_*m*_ determines the profile of *W*_*ij*_ and the scale of the grid firing patterns in a neural sheet. According to the 3σ rule in probability distribution

$$\begin{array}{l} T_{m}\approx R_{w}/2\approx 3*\sigma \;,\;T_{m}\approx N/n_{m} \end{array}$$

where *R*_*w*_ is the radius of the grid module's weight profile and *T*_*m*_ is the grid period of the *m*th grid module, as shown in Figures 3B,C. So $\sigma_{m}\approx \frac{N}{3*n_{m}}$. Thus σ_*m*_ can be approximated as:

(10) $$\begin{array}{l} \sigma_{m}\approx \frac{N}{c*n_{m}} \end{array}$$

In practice, the value of *c* needs some fine-tuning in a range from 3 to 4 for high-quality hexagonal firing patterns of grid cells.

**Figure 5:**
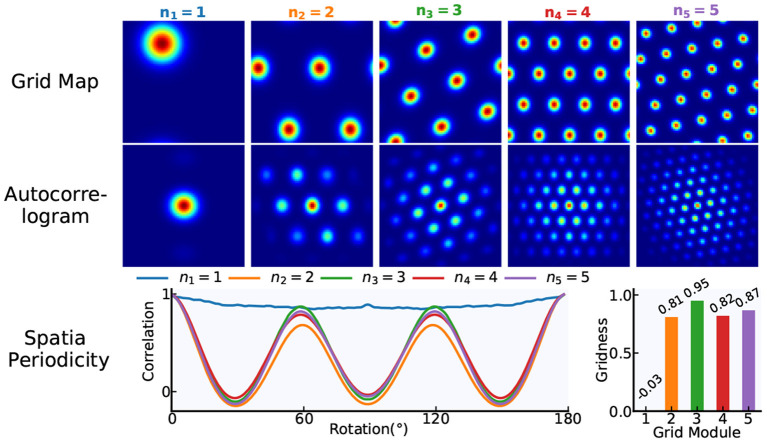
The gridness of grid cells. Based on our proposed modeling mechanism, a multi-scale grid model including five modules is taken as an example and the network size of each module is *N* × *N* = 84 × 84. According to the gridness measure methods, the gridness of each module in the model is quantified.

Grid cells are characterized by the periodic spatial distribution of hexagonal firing patterns. By calculating the “Gridness” of grid cells' spatial firing patterns, the six-fold rotational symmetry in the firing patterns is quantified. From the firing rate map, we can derive the spatial autocorrelogram and the annulus containing six peaks nearest the origin. For the annulus and its rotated version, grid cells with high-quality firing patterns should have a greater correlation for 60° and 120° rotation than for 30°, 90°, and 150° rotation. Then, the “Gridness” measure is calculated as the minimal 60° and 120° correlation minus the maximum of 30°, 90°, and 150° correlation. Taking *N* = 84 as an example, the gridness of grid cells in our model is illustrated in Figure 5.

Different from the single-scale grid cell model in Burak's work, fine-tuning σ_*m*_ and regularly adjusting *w*_0_ will achieve grid cell models with different spatial scales and network size for path integration. Specifically, grid cells with different scales can be modeled by fine-tuning σ_*m*_ in the case of the same network size (Figure 3E), and grid cells with a similar spatial scale can be modeled by regularly adjusting *w*_0_ in the case of different network size (Figure 4C). Despite many similar works on grid cell modeling based on CAN, in this paper, the grid cell modeling mechanism we proposed for path integration is easy to use and extend, making parameter setting about the multi-scale extension of grid cell model more convenient, effective, and repeatable.

### 2.3. Learning to Topologically Represent Space

Place cells were considered as the first convincing demonstration (O'Keefe and Dostrovsky, 1971) of a cognitive map, and early computational models about place cells were constructed for path integration (Samsonovich and McNaughton, 1997; Conklin and Eliasmith, 2005). The discovery of grid cells in MEC indicates that path integration is most likely performed by grid cells (Fyhn et al., 2004a; Hafting et al., 2005; McNaughton et al., 2006). One grid cell will fire at different locations, and different grid cells may fire at the same location. Then the combinatorial coding of grid cells with multiple spatial scales can form a unique identity for each spatial position. Place cells are suggested to be one synapse downstream of grid cells (Fyhn et al., 2004b; Hafting et al., 2005), which provide animals with an efficient way of encoding spatial information. Combining grid cells with place cells achieves path integration in space.

**Figure 6:**
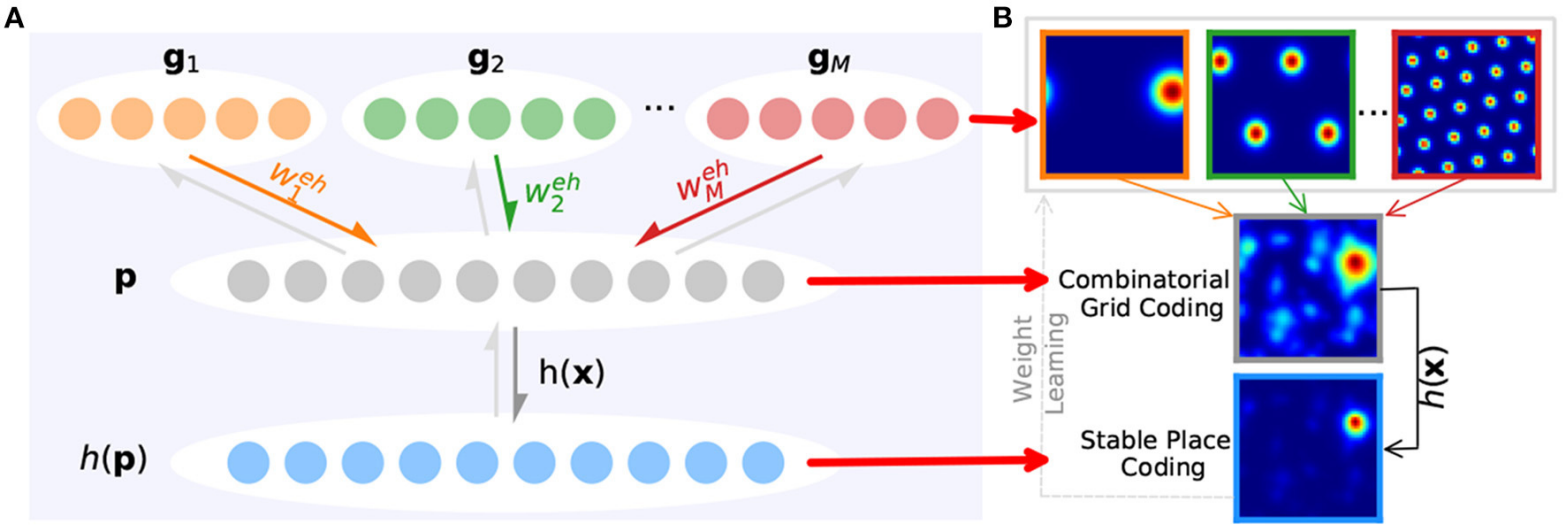
Learning stable spatial representation from grid cells' path integration. **(A)** Taking the multi-scale grid cells' path integration as upstream of place cells (colored arrows) and *h*(***x***) as the activation function (gray arrow), place cells can encode the space and bring feedback to weight training (light gray arrows). **(B)** First row: grid cells' firing rate maps with multiple scales. Second row: the combinatorial firing rate of grid cells. Third row: place cells' firing rate map.

Generally speaking, connections between grid cells and place cells are built through a feedforward network with full connections between layers, which is a bit different in this paper. According to the path integration mechanism in the CAN-based grid cell model, the path integration in space can be derived from the smooth movement of the grid cell population's activity bumps in grid neural sheets. When it comes to connecting to place cells for spatial representation, grid cells with the same scale in a grid module can be considered as a whole that shares the same weighted connections to place cells, as shown in Equation (11) and Figure 6. That is, connection weights between all grid cells in a grid neural sheet and place cells are the same, and weight values needing to be learned are equal to the number of grid cell modules.

(11) $$\begin{array}{l} p_{i}=h\left(\overset{M}{\underset{j=1}{\sum }}w_{j}^{eh}g_{j}\right) \end{array}$$

(12) $$\begin{array}{l} h\left(x\right)=1/\left(1+e^{k_{p}\left(x-max\left(x\right)\right)}\right) \end{array}$$

where the number of grid modules is denoted as *m* as above and $w_{j}^{eh}$ is the synaptic weight from the *j*th grid module to the place cell module. *k*_*p*_ affects the number of peaks of place cell population activities. The place cell activation *h*(***x***) can transform the weighted sum of grid cell activities into their exponent's product, then the activity of place cells obtaining higher-level overlapping grid cell activities will be magnified to a larger extent, which helps select a single activated place cell for place encoding at a time.

For generating spatially specific patterns from the periodic activity of grid cells without phase alignment, grid activity—as the basis of place cell activity—should be appropriately weighted (Savelli and Knierim, 2010; Zeng and Si, 2017). Place cells learn to take an appropriately weighted sum of grid cell population activity as input. As the environment has been extensively explored, the place cell activity can be refined by learning (as shown in Figure 6) and a map that reflects the environmental topology can be built in which place cells are constructed as nodes in the map. To compute place cell population activity, competitive Hebbian learning is generally used, which can be extended to the temporal difference (TD) version in a more biologic way. For the excellent nature embedded in our proposed grid cell and place cell models, variant unsupervised learning rules can be used to achieve stable place representation of place cells. Here we chose the following:

(13) $$\begin{array}{l} dw_{i}^{eh}/dt=lr*{\text{p}}_{i}*{\left({\text{g}}_{i}-{\text{p}}_{i}*w_{i}^{eh}\right)}^{⊤} \end{array}$$

where *lr* is the learning rate. The learning rule contributes to the gradually reliable place representation of place cells with the robot's further exploration of the environment, as shown in Figure 6. In addition, we constrain $w_{i}^{eh}\geq 0$ and $\overset{m}{\underset{j=1}{\sum }}{\left(w_{j}^{eh}\right)}^{2}=1$ to prevent weights becoming negative or some neurons always winning competition (Rolls et al., 2007). The sum of all weights is kept constant, and small weights will be reduced by weight competition.

**Figure 7:**
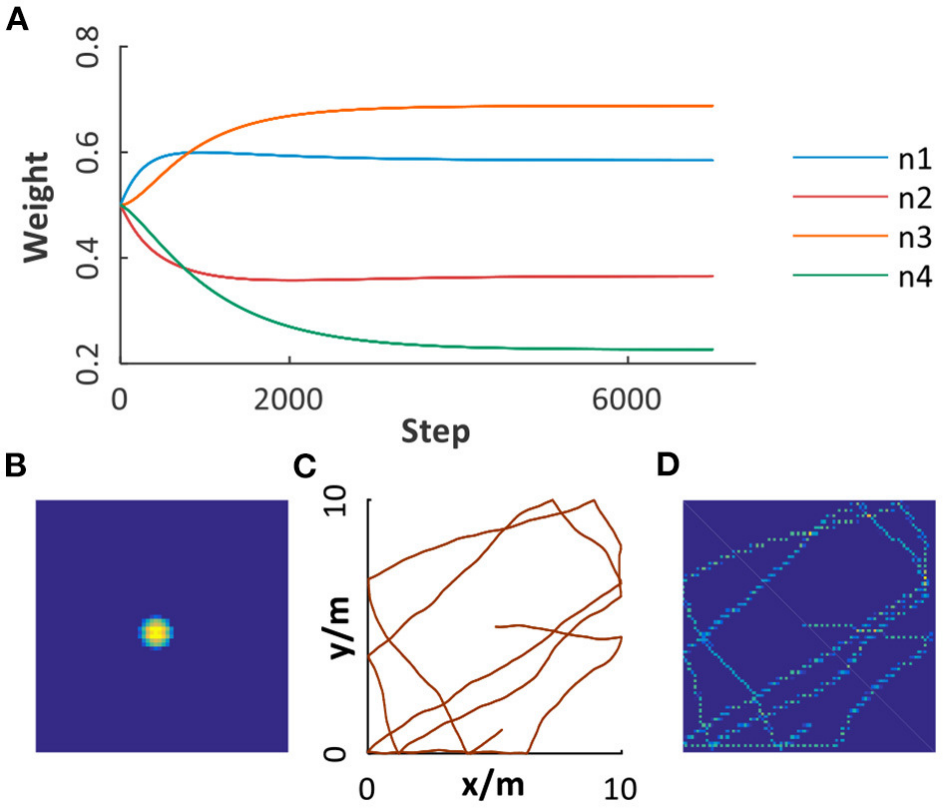
Path integration based on grid cells and place cells. **(A)** The weights learned from grid cell modules with multiple scales to place cell module. **(B)** The place field of a place cell. **(C)** A trajectory in a 10*10*m*^2^ open area. **(D)** Path integration result illustrated by the activation of place cells encoding corresponding spatial positions.

Before map building for the environment, the robot will randomly explore the environment for data collection and learning between grid cells and place cells. As illustrated in Figure 7A, synaptic weights between place cell modules and grid cell modules with four spatial scales are taken as an example. We can see that the learning process converged after 4, 500 steps, contributing to the ideal firing pattern of place cell (Figure 7B) and stable place encoding. Figures 7C,D, respectively illustrate a trajectory in a 10*10*m*^2^ open area and the encoding results of place cells, in which stable and consistent path integration is achieved. Moreover, the ability to learn to topologically represent the environment embedded in place cells can work well in different environments, which will be verified in the following experiments.

### 2.4. Hierarchical-Structure-Based Visual Template Matching for Real-Time Loop Closure Detection

Evidence has revealed that when a rat returns to a familiar environment, the inner path integrator would be reset to adjust to the perceived environment (Fuhs and Touretzky, 2006; Moser et al., 2008). However, it remains unclear how the brain perceives and transforms external sensory cues into partial signals of the internal cognitive map. In map building and navigation based on a mobile robot, external sensory inputs, especially visual cues, are often used for place recognition and loop closure detection. During the map-building process, fast visual scene matching can facilitate the whole system's real-time performance. Inspired by the hierarchical characteristic of a tree structure, we proposed a novel visual template organization method that speeds up the visual scene matching and loop closure detection process.

Each visual scene is represented by a pair of images, including RGB and depth information. Depth information helps to relieve ambiguity caused by RGB images and is less sensitive to lighting conditions (Lai et al., 2011). By calculating the difference between two visual scenes, we can determine whether they match or not (Tian et al., 2013), as shown in the following formula:

(14) $$\begin{array}{l} d=W_{r}*d_{r}+W_{d}*d_{d} \end{array}$$

where *d*_*r*_ is the difference of RGB information, *d*_*d*_ is the difference of depth information, and *W*_*r*_ + *W*_*d*_ = 1. If *d* is larger than the scene-matching threshold *M*_*t*_, the two scenes are recognized as different scenes. Otherwise, they are considered as the same scene. It should be noted that to compute *d*_*r*_ and *d*_*d*_, visual features can be obtained in many ways, such as Scale-Invariant Feature Transform (SIFT), Convolutional Neural Network (CNN), and so on.

In our system, visual scenes are classified as different visual templates and stored in a visual template library for loop closure detection. When a visual template matching the current scene is found, a loop closure can be detected for path integration error correction. Otherwise, a new visual template for the current scene will be created and stored in the visual template library.

**Figure 8:**
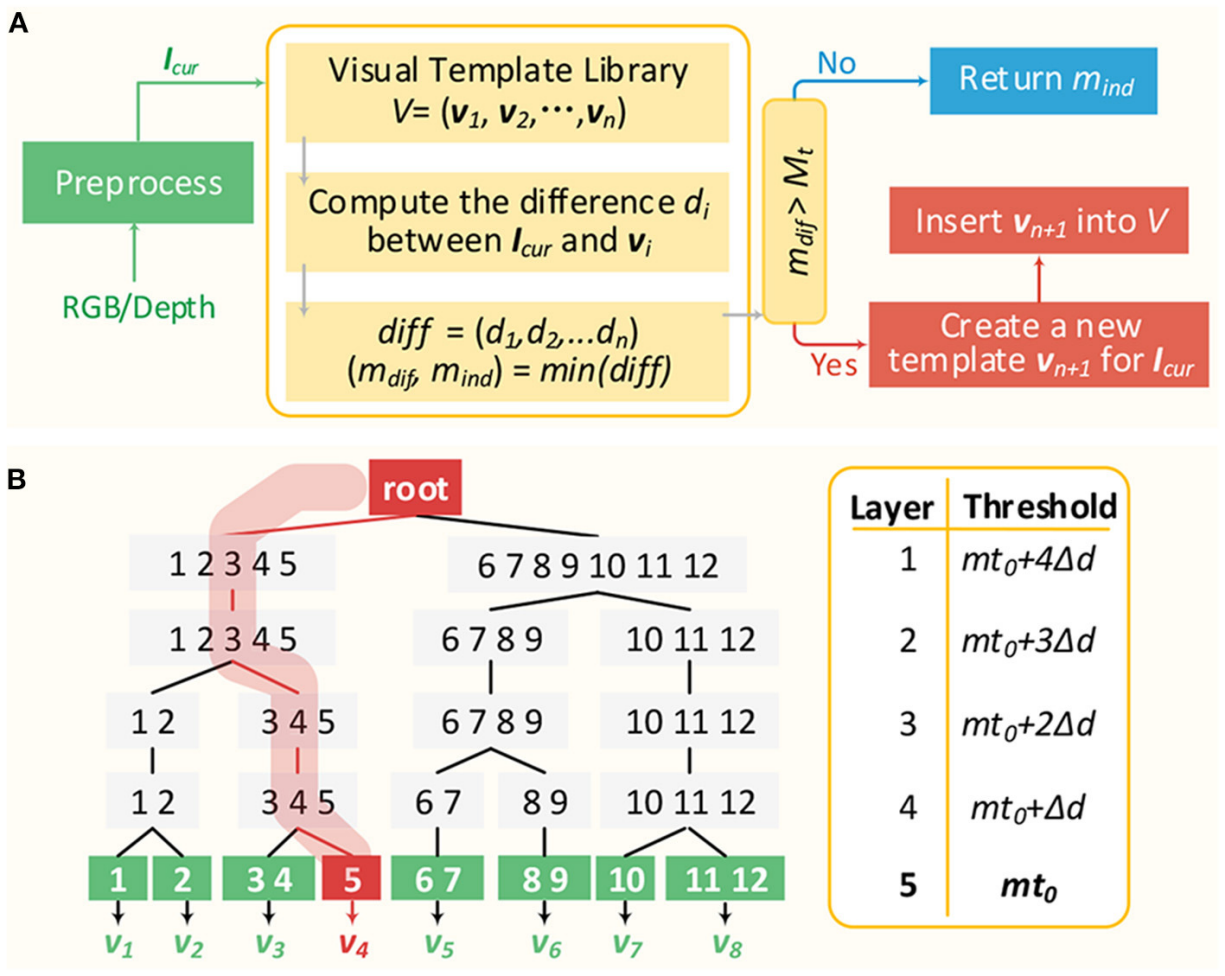
Visual template matching. **(A)** The typical visual template matching process. **(B)** An simple example for illustrating VTT mechanism.

The typical process of visual template matching is described in Figure 8A. Firstly, the RGB and depth image of the current scene captured from RGB-D sensor are preprocessed as *I*_*cur*_, representing image features. Secondly, the differences between *I*_*cur*_ and all templates in visual template library are calculated, respectively. Then the minimum difference *m*_*dif*_ and the corresponding visual template *v*_*m*_*ind*__ are selected out. If *m*_*dif*_ is larger than *M*_*t*_, then a new visual template *v*_*n*+1_ representing the current scene will be created and inserted into the visual template library. Otherwise, the current scene will be considered being the same scene as *v*_*m*_*ind*__, indicating that a loop closure is detected. It can be seen that every time a visual scene comes, the differences between it and all templates in the library need to be obtained, putting stress on the real-time performance. In previous works (Tian et al., 2013; Yuan et al., 2015), visual templates were simply linearly organized and retrieved one by one until a matching template is found. Then the matching time grows linearly with the number of templates and the real-time performance is severely impacted.

According to the above matching rule, the visual scene matching can be done at different levels of granularity by changing the matching threshold. A larger threshold *M*_*t*1_ indicates more relaxed matching criteria (coarse granularity) and a smaller threshold *M*_*t*2_ indicates more restricted matching criteria (fine granularity). With *M*_*t*1_, two visual scenes with larger differences may be considered to match each other. While with *M*_*t*2_, the two scenes may be recognized to not match. In this paper, the concept of multi-granularity matching is transformed into a novel hierarchical top-down visual template tree (VTT). It can relieve the visual template matching stress since the visual scene matching time performance is mainly related to the tree depth, not the number of templates in the library.

Figure 8B illustrates a simplified VTT with 5 layers (*l*_1_ − *l*_5_). Values in the right-hand table are the corresponding matching thresholds of layers, where *mt*_0_ is the optimal matching threshold used to determine whether two visual scenes match each other or not and △*d* is the threshold difference between layers. Three kinds of nodes are included. The root node is just as the root of the tree without any data. Non-leaf nodes (*l*_1_ − *l*_4_) are responsible for classifying the current visual scene into the correct child node of the next layer. Leaf nodes (*l*_5_) represent visual templates in the visual template library. Additionally, *v*_*i*_ is denoted as the visual template belonging to the corresponding leaf node. Every time a visual scene *I*_*cur*_ arrives, and the matching with nodes in *l*_1_ will firstly be done according to *M*_*t*_ = *mt*_0_ + 4△*d*. If so, then consider the following:

1. If the minimum matching difference (*m*_*ind*_) is smaller than or equal to *mt*_0_ + 4△*d*, then *I*_*cur*_ will be classified into the *m*_*ind*_ node. Additionally, the matching with children of the node *m*_*ind*_ in *l*_2_ continues with matching threshold *M*_*t*_ = *mt*_0_ + 3△*d*.
2. If the minimum matching difference with nodes in *l*_1_ is larger than *mt*_0_ + 4△*d*, then a new tree branch rooted from the root is created for *I*_*cur*_ (representing a new visual template).

This process is repeated until a new leaf node (a new template) is created for *I*_*cur*_ or it is classified into a leaf node in *l*_5_.

VTT has a comparative advantage in real-time visual scene matching since its time performance is mainly related to the tree depth and does not linearly increase with the number of visual templates. It is not noticeable when the visual template library has a small capacity, while with further environmental exploration and visual template accumulation, VTT can contribute to considerable retrieval time reduction compared with a linearly organized visual template library.

### 2.5. Cognitive Map Building

By combining path integration of multi-scale grid cells, place encoding, and visual scene matching, the cognitive map building can be done for obtaining the topological structure of the environment. Through the path integration of grid cells and place encoding of place cells, the robot's spatial location can be represented. Nodes in the cognitive map are constructed by associating these spatial locations with corresponding visual cues and denoted as cognitive experiences. Algorithm 1 describes the cognitive map building process, which shows how a robot can build a topological map for the environment by integrating self-motion and sensory information. The incoming visual inputs are compared with historical visual scenes related to corresponding cognitive experiences. The latest input will be compared with previous cognitive experiences based on spatial distance and vision. If it matches one of the previous experiences, it will be considered as a familiar scene that had been seen previously by the robot. The status of the grid cell and place cell population activities are then reset to the previous matched cognitive experiences. The current visual input and the matched cognitive experience are merged into one experience. Otherwise, a new cognitive experience is created. Once a loop closure is detected, the map will be corrected to adjust to the recalled cognitive experiences.

**Algorithm 1:** Cognitive Map Building

**Input** Raw odometry data from the robot wheel encoders and visual images from the RGB-D sensor
**Output** Cognitive map
**Begin**
Calculate grid cell population activities using Equations (1–4)
Calculate place cell population activities using Equations (7–8)
Obtain the current activated place cell
Perform visual template matching
**if** The incoming visual scene matches previous cognitive experiences **then**
Perform resetting and map correction
**else**
Create a new visual template for the current scene
Add a new experience node to the cognitive map for the current scene
**end if**
**End**

### 2.6. Grid Cells and Motion Planning

Because of their unique spatial firing patterns, a relatively small number of grid cells can code an animals' location across a large range of territory. In this section, the coding advantage of grid cells in motion planning is demonstrated by achieving grid-cell-based multi-scale motion planning. With the further application of robots in daily life, motion planning in robotics has always been a major problem. The objective of motion planning is to plan available paths from the start position to the target region, given the start and target configuration of the robot. Many motion planning algorithms have been developed for meeting the increasing robotic application requirement. Rapidly-exploring Random Tree (RRT) is a popular algorithm, a sampling-based stochastic searching method, and RRT^*^ was introduced as an optimal variant of RRT (Karaman and Frazzoli, 2011). In RRT^*^, an initial path is first identified and then improved upon by re-wiring the samples, replacing old parents with new ones whose cost is less, according to the Euclidean distance from the initial state.

However, it has very slow convergence rates to the optimal solution in cluttered environments and high dimensional spaces, and many RRT^*^ variants have been developed. In this section, we integrate grid cell activity into RRT^*^ to achieve multi-scale motion planning, called GC-RRT^*^ methods. The integration algorithm GC-RRT^*^ is illustrated in the pseudocode of Algorithm 2.

**Algorithm 2:** GC-RRT*

*GC*_*set*_ ← Select_Activecell(*q*_*init*_, *q*_*goal*_, *GC*_*patterns*_)
**for** *g*_*j*_ ∈ *GC*_*set*_ **do**
*V* ← {*q*_*init*_}, *E* ← ∅
*g*_*scale*_ ← the grid scale of *GC*_*patterns*_[*g*_*j*_], *GP*_*set*_ ← Get_Activepos(*GC*_*patterns*_[*g*_*j*_])
**for** *i* = 1, ⋯, *Iter* **do**
*q*_*rand*_ ← Sampling(*GP*_*set*_)
*q*_*nearest*_ ← Nearest(*G*=(*V*, *E*), *q*_*rand*_)
*q*_*n*_*ew* ← Steer(*q*_*nearest*_, *q*_*rand*_)
**for** ObstacleFree(*q*_*nearest*_, *q*_*new*_) **do**
*Q*_*near*_ ← Near(*G*=(*V*, *E*), *q*_*new*_, γ*g_*scale*_)
*V* ← *V*∪{*q*_*new*_}
*q*_*min*_ ← *q*_*nearest*_, *c*_*min*_ ← Cost(*q*_*nearest*_) + *c*(*q*_*nearest*_, *q*_*new*_)
**for** *q*_*near*_ ∈ *Q*_*near*_ **do**
if CollisionFree(*q*_*near*_, *q*_*new*_) ∧ Cost(*q*_*near*_) + *c*(*q*_*near*_, *q*_*n*_*ew*) < *c*_*min*_ **then**
*q*_*min*_ ← *q*_*near*_, *c*_*min*_ ← Cost(*q*_*near*_) + *c*(*q*_*near*_, *q*_*new*_)
**end if**
**end for**
*E* ← *E*∪{(*q*_*min*_, *q*_*new*_)}
**for** *q*_*near*_ ∈ *Q*_*near*_ **do**
if CollisionFree(*q*_*new*_, *q*_*near*_) ∧ Cost(*q*_*new*_) + *c*(*q*_*new*_, *q*_*near*_) < Cost(*q*_*near*_) **then**
*q*_*parent*_ ← Parent(*q*_*near*_)
*E* ← (*E*\{(*q*_*parent*_, *q*_*near*_)}) ∪ {(*q*_*new*_, *q*_*near*_)}
**end if**
**end for**
**end for**
**end for**
**return** *G*_*j*_=(*V*, *E*)
**end for**
*P*_1_, ⋯*P*_*j*_, ⋯ = Rewiring(*G*_1_, ⋯, *G*_*j*_, ⋯)
*P*_*optimal*_ = Select_minCost(*P*_1_, ⋯*P*_*j*_, ⋯)

Compared with the original RRT^*^, under the same spatial resolution, GC-RRT^*^ can not only provide a multi-scale path planning mechanism but can also achieve a greater chance of success for the agent's path planning, as shown in the following experiment.

## 3. Results

### 3.1. Robot Platform and Datasets

**Figure 9:**
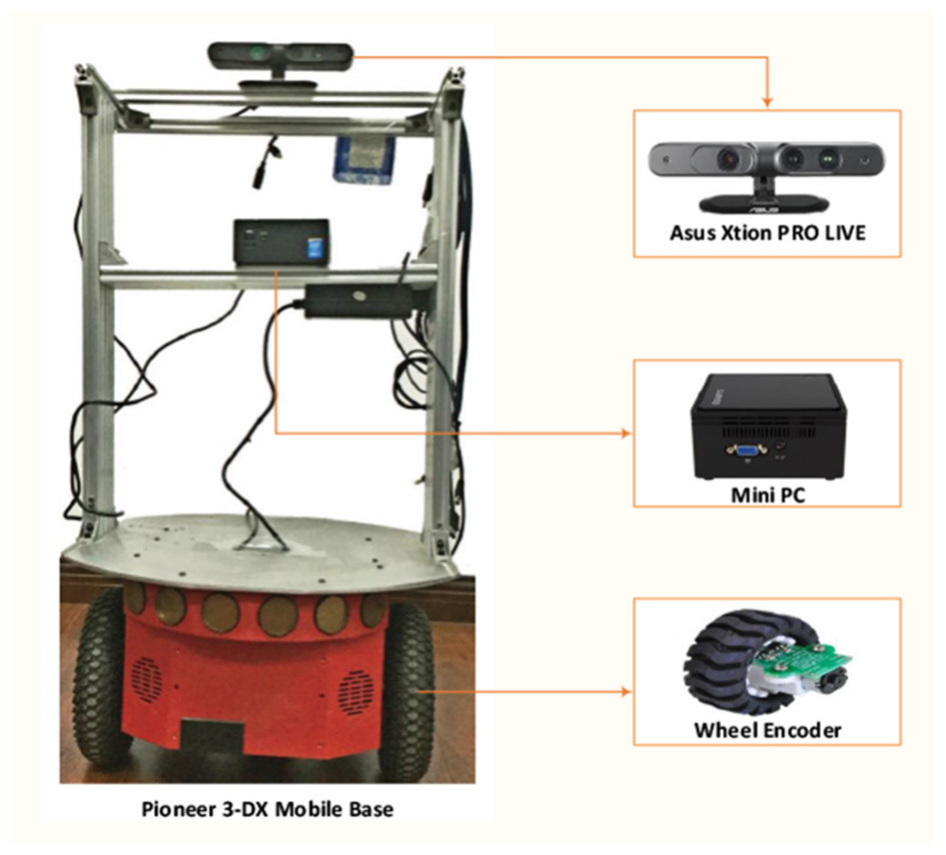
Robot platform.

As illustrated in Figure 9, our system is implemented on a mobile robot platform, which consists of a Pioneer 3-DX mobile base, an ASUS Xtion PRO Live (RGB-D sensor), and a MiniPC. The mobile base consists of two front wheels with encoders recording the self-motion information and a rear wheel for stabilization. The Xtion PRO Live is mounted on the front-top of the Pioneer 3-DX mobile base to capture RGB-D images of the current scene in real time.

The datasets used in experiments are listed below:

1. *NCRC Lab* datasets, two indoor datasets, respectively, with 939 and 1153 pairs of images (RGB and depth). They are gathered from a laboratory with images recorded by the RGB-D sensor of the above robot platform which is remotely controlled to do semi-automatic environmental exploration.
2. *Singapore Office* dataset (Tian et al., 2013), a large office (35 × 35*m*^2^) dataset with 2110 pairs of images (RGB and depth) captured by a RGB-D sensor.
3. *QUT iRat 2011 Australia* dataset (Milford and Wyeth, 2008), an indoor dataset with 16658 RGB images obtained when an iRat explored a road movie set based on Australian geography.
4. *Oxford New College* dataset (Milford and Wyeth, 2008), a university campus dataset with 7855 panoramic images (left and right) captured from Oxford University.

### 3.2. Place Representation

**Figure 10:**
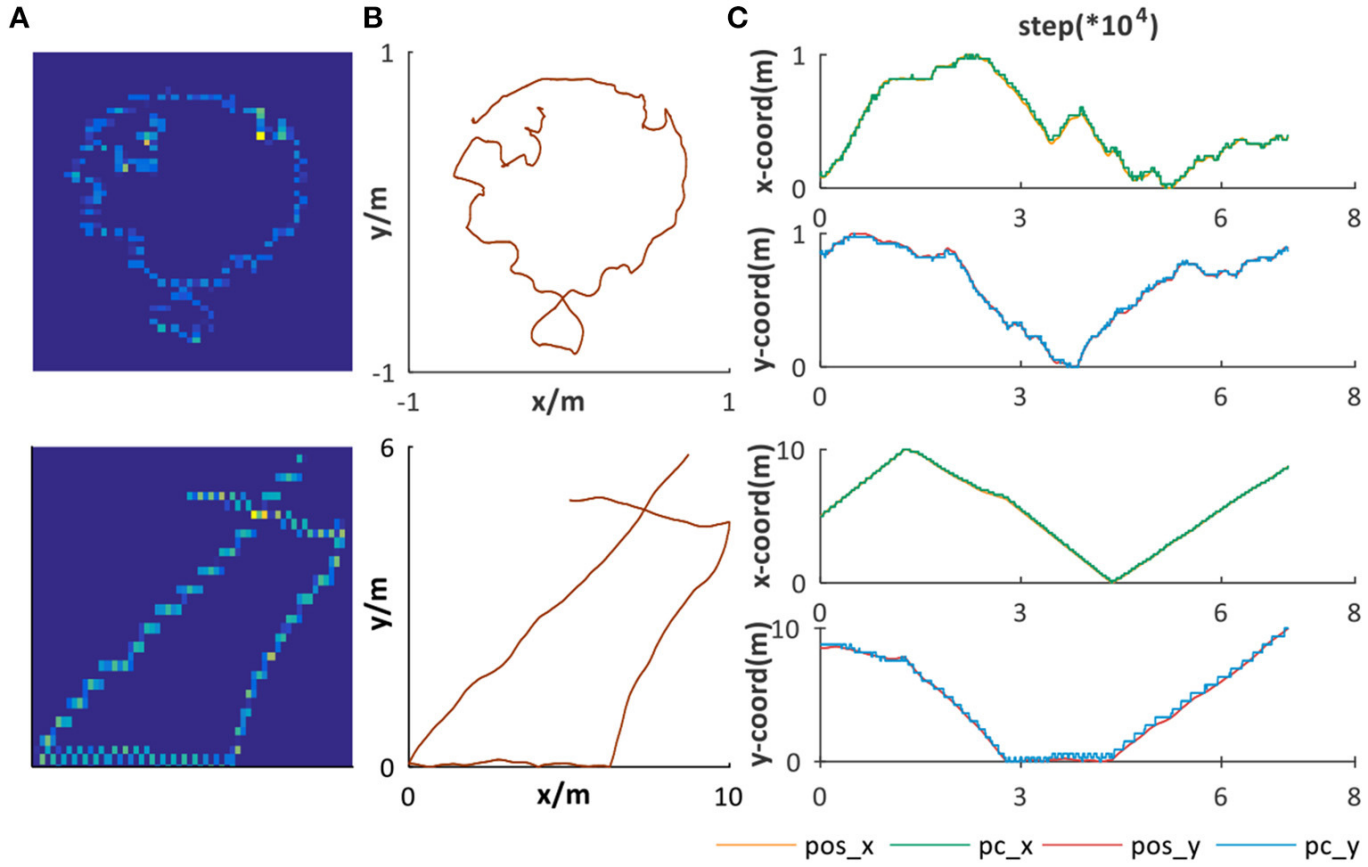
Place representation. **(A)** Path integration result, illustrated by the activation of place cells encoding corresponding spatial positions. **(B)** The ground truth trajectories. **(C)** The comparison between ground truth and the spatial encoding result. *pos*_*x* and *pos*_*y* are, respectively, the *x* and *y* coordinates' sequences of **(B)**. *pc*_*x* and *pc*_*y* are, respectively, the *x* and *y* coordinates' sequences of **(A)**.

In our system, the path integration result of velocity-driven grid cells is converged into place cells for place representation. Two datasets are chosen as ground truth for verifying the place representation performance. The first one is from Hafting et al. (2005) in a 1 × 1*m*^2^ open area and the second one is randomly simulated in a 10 × 10*m*^2^ open area, as shown in Figure 10B. Taking linear and angular velocity information of them as input, our system can successfully obtain the corresponding trajectories, as shown in Figure 10A.

To further verify the stable and accurate path integration and place representation performance of our system, we compared the *x* and *y* coordinates of Figures 10A,B, respectively, as illustrated in Figure 10C. The spatial encoding results have great alignment with the ground truth in both small and large areas. To summarize, the place representation mechanism we proposed based on offline unsupervised learning rule can not only form effective place encoding but also is a general solution for different environments, which are indispensable characteristics for dead reckoning and successful cognitive map building.

### 3.3. Dead Reckoning

Dead reckoning is a spatial navigation ability based on the inner path integrator of rodents without vision assistance. Similarly, to simulate the dead reckoning ability of our system, the mobile robot platform is required to only depend on odometry from wheel encoders as the single input into the path integration part and the whole process only depends on the spatial cells' location memorization ability and without external vision's aids.

**Figure 11:**
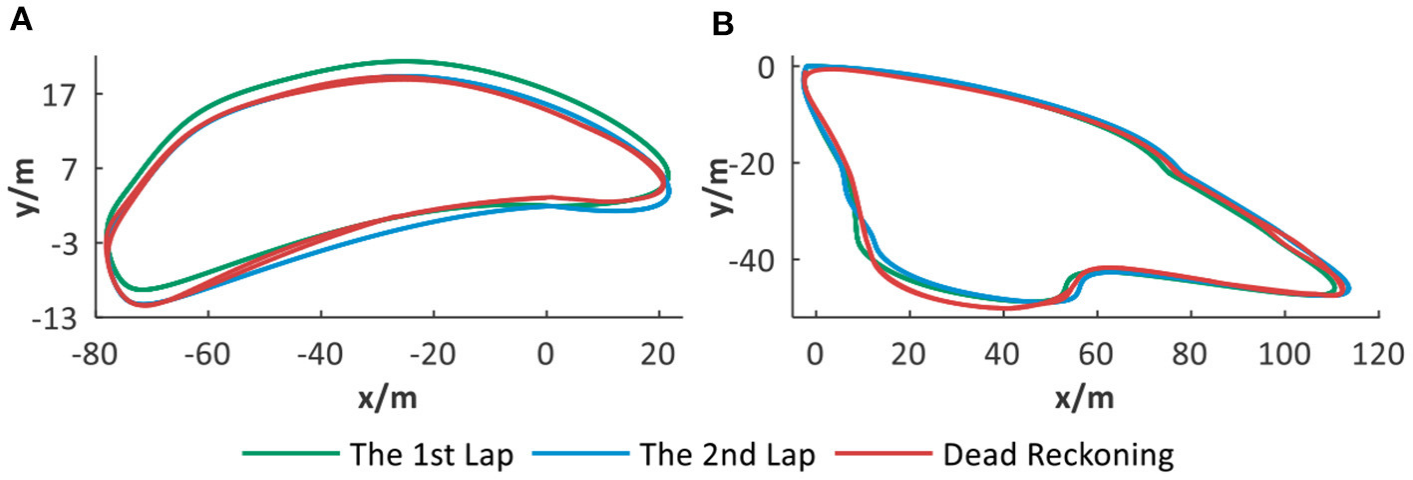
The dead reckoning ability analysis. **(A,B)** Different trajectories with drift and corresponding dead reckoning results based on spatial cells.

Two noisy trajectories are simulated and used for evaluating the dead reckoning ability without vision assistance. Each trajectory includes two laps with drift. As displayed in Figure 11, our system can correct trajectory drift and build a cognitive map. It is indicated that dead reckoning ability can be acquired through the spatial memorization ability of spatial cells. When the robot visits the same place again, successful localization can be achieved through activating the corresponding place cell. The system can take advantage of the space memorization ability of place cells to correct part of position drift and implement relatively effective localization.

The dead reckoning ability evaluation is quantified and illustrated in Table 1. *M*_1_ and *M*_2_ are denoted, respectively, as the number of cognitive map experiences created for the first and second laps of trajectory and *M*_*t*_ = *M*_1_ + *M*_2_. *M*_*p*_ and *M*_*r*_ are defined as below:

1. *M*_*p*_ : the number of positions localizing to history experiences during the second lap of trajectory.
2. *M*_*r*_ = (*M*_*p*_ / *M*_1_) × 100%, reflecting the place cells' space memorization ability.

**Table 1:** Statistical results for dead reckoning.

	***M*_*t*_**	***M*_1_**	***M*_2_**	***M*_*p*_**	***M*_*r*_**
1	241	172	69	116	67.44%
2	232	159	73	113	71.06%

Table 1 shows the statistical results during the dead reckoning based on the above two simulated trajectories. Taking the first simulated trajectory as an example, 67.44% of place cells representing history experiences are recalled for localization during the second lap. Therefore, with drift in a certain range, our system can achieve quite good dead reckoning performance. However, for large fluctuation of trajectory drifts, it is hard to keep acceptable performance. External assistance is needed for loop closure detection and localization error correction in a cognitive map, such as visual cues.

### 3.4. Visual Scene Matching

**1) Time Performance Analysis**

Three datasets are used for evaluating the real-time matching performance of our proposed VTT method. Keeping the same parameter settings and image similarity computation methods, we compared the time performance with linearly organized structure (Milford and Wyeth, 2008; Tian et al., 2013).

**Figure 12:**
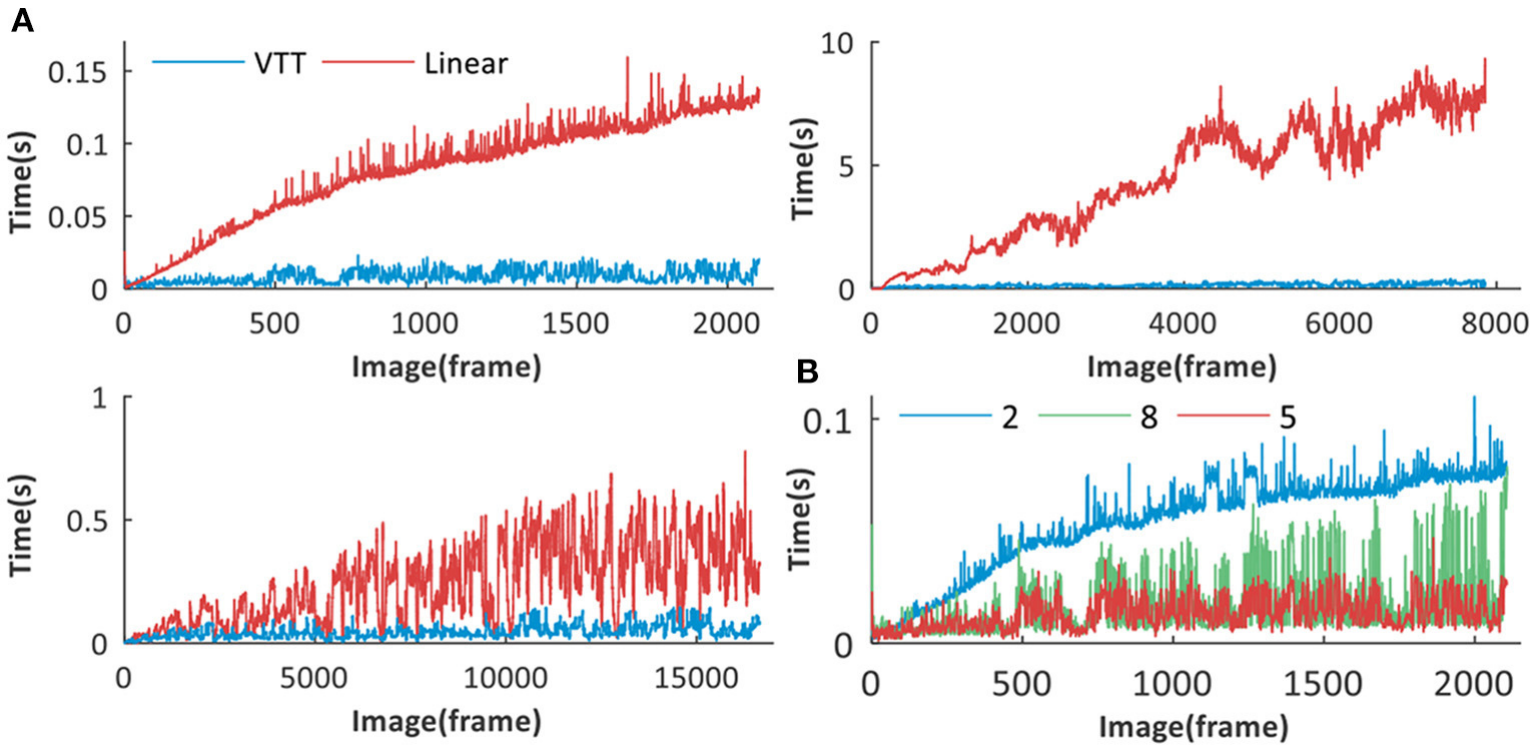
Time performance analysis of VTT. **(A)** Time performance comparison between VTT and linear structures for Different datasets: *Singapore Office, QUT iRat 2011 Australia* and *Oxford New College*. **(B)** Time performance comparison between visual template matching based on VTT with different number of layers (2, 5, 8).

It can be seen from Figure 12A that, with the rapid accumulation of visual templates, VTT is much better than the linear structure. For the latter, much more time is spent on retrieving visual templates one by one to find matching ones. While because of VTT's superiority in visual template organization, the time spent on visual template matching keep stable in a certain range. It is important to emphasize that our proposed VTT has stable time performance even with a large visual template library so that it would have tremendous application potential in large environments.

The number of VTT layers will affect the time performance in the visual scene matching. Taking the *Singarpore Office* dataset as an example, we create VTT with different sizes of layer (8, 5, and 2) for visual scene matching. The matching time comparison is shown in Figure 12B and the total matching time is calculated, respectively 42.62, 19.29, and 113.96 s. It can be seen that 5 layers (excluding the root layer) can obtain the best time performance.

**2) Scene Recognition Performance Analysis**

**Figure 13:**
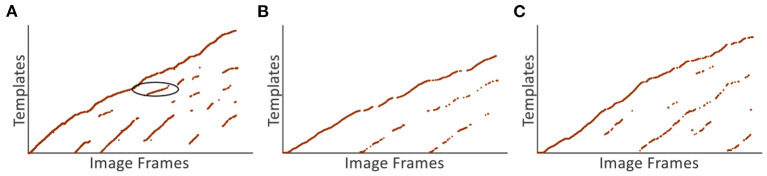
Scene recognition results based on VTT. **(A)** The scene recognition result of the *Singapore Office* dataset. **(B,C)** The scene recognition results of *NCRC Lab* datasets.

In this section, the scene recognition performance based on VTT is analyzed. Experiments are firstly done on the *Singapore Office* dataset and Figure 13A demonstrate the scene recognition results based on VTT. The *x*-axis and *y*-axis represent image frames and corresponding visual templates, respectively. More regular point distribution and fewer noisy points mean better visual template matching performance, e.g., the point distribution in red circle regions. It means the current scene is stably matched with existing templates in the visual template library.

**Figure 14:**
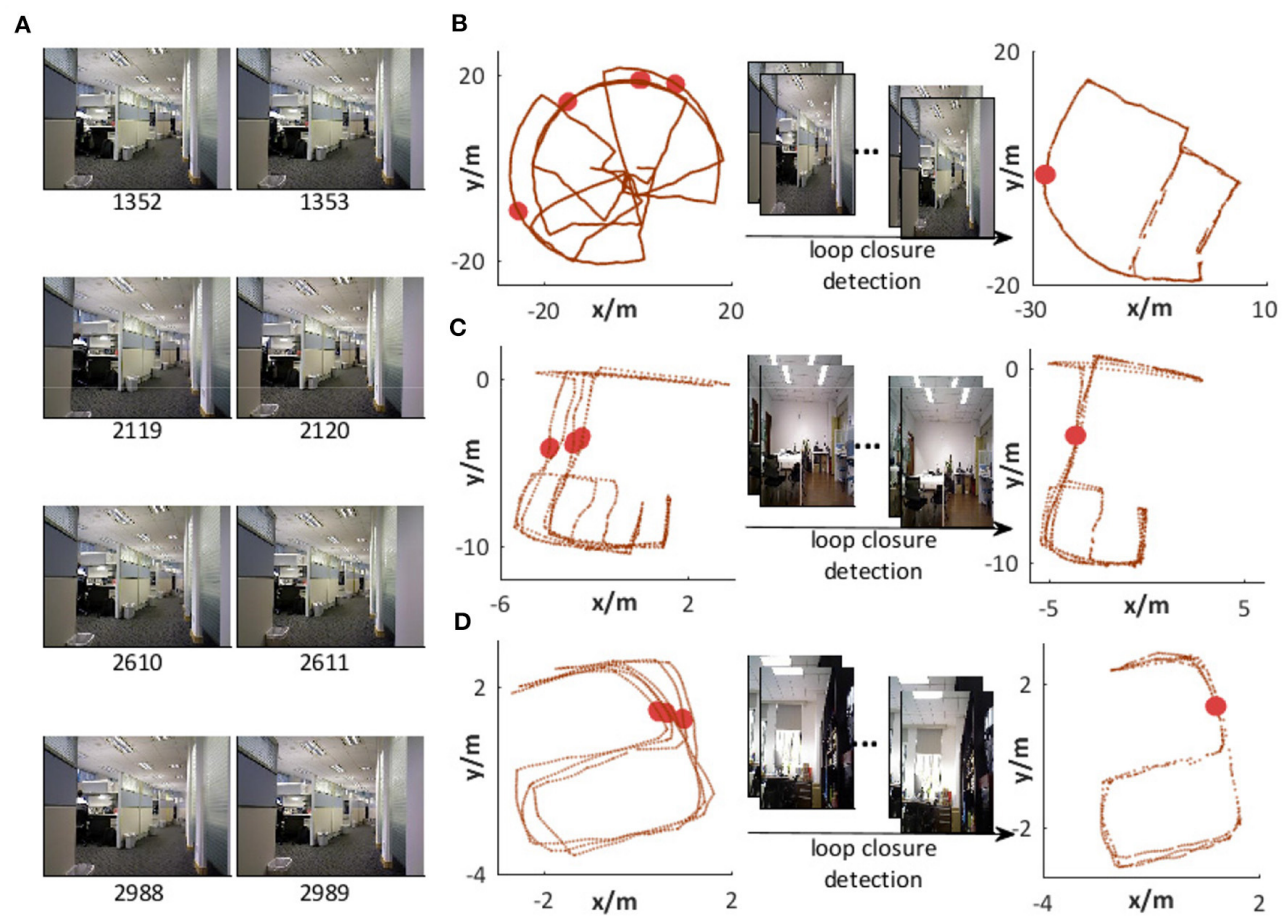
Scene recognition. **(A)** Images recognized as being in the same place. **(B)** Loop closure detection with visual assistance on the *Singapore Office* dataset. **(C,D)** Loop closure detection with visual assistance in our lab environment.

Figure 14A shows a group of images recognized as being in the same place, i.e., all of them match a specific template in VTT. It can be seen from Figure 14B that the valid loop closure detection can be executed based on right scene recognition. The accumulated odometry error from wheel encoders leads to different space coordinates in the same place, as shown on the left-hand side of Figure 14B. With the assistance of loop closure detection based on scene recognition, the robot can correct errors, as shown on the right-hand side of Figure 14B. We also test the scene recognition performance on two other datasets from *NCRC lab*. The scene recognition results are illustrated in Figures 13B,C. Figures 14C,D demonstrate that visual scenes that are physically near to each other and should belong to the same place can be successfully considered as being in the same place.

However, less noise does not always mean that it can assist the system in achieving the right loop closure detection and better map correction. Therefore, we should further verify the complete performance by introducing it into the system for building a cognitive map.

### 3.5. Vision-Assisted Cognitive Map Correction

In this experiment, the dead reckoning based on spatial cells and the visual template matching based on VTT are combined to form the system architecture as shown in Figure 1. Table 2 shows the parameter setting and the map building performance of the whole model is also analyzed.

**Table 2:** Parameter setting.

**Parameter**	**Setting**
Shift in outgoing weights *l*	2
Layers of neural sheet	4
Size of neural sheet N^*^N	40^*^40
Time-constant of neuron response τ	5 ms
Learning rate *lr*	0.0001

**Figure 15:**
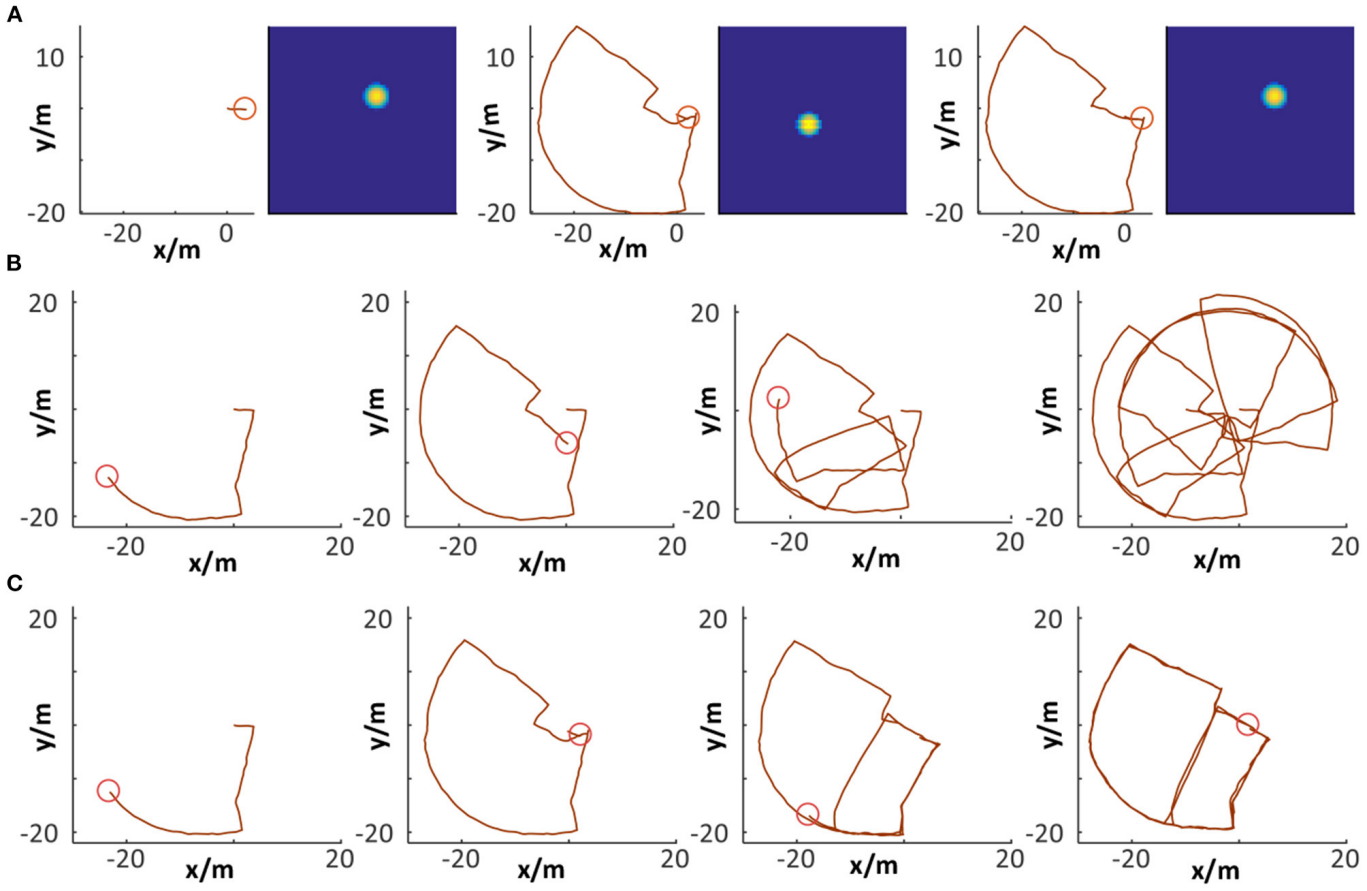
Cognitive map building. **(A)** Loop closure detection and path integration resetting. **(B,C)** Comparison between raw odometry and cognitive map.

Several states of place cells and the corresponding cognitive map are captured during the map-building process based on the *Singapore Office* data. In Figure 15A, a specific position (the red circle) and the corresponding activated place cell are illustrated in the first column. The second column describes the moment when loop closure is detected with the VTT assistance but neuronal activities are not reset. The system does map correction and neuronal activities are reset in the third column and the correspondence between position and activated place cell is achieved, which is the same as the first column.

Comparisons between raw odometry and cognitive maps have been done, as shown in Figures 15B,C. The former shows the raw trajectory obtained from the robot wheel encoders at four specific moments. It cannot properly represent the environment map because of accumulated errors. The latter shows the corresponding cognitive map built by our system. With dead reckoning of spatial cells and VTT-based visual scene matching, loop closure detection can be successfully performed and the accumulated error can be corrected. Finally, a cognitive map is generated that encodes both topological and metric information of the environment. It is observed that before the first loop closure was detected, the cognitive map was the same as the raw odometry trajectory. When the loop closure was detected (i.e., the robot detected a scene which it had experienced), cell activity resetting is performed, and the map is corrected.

**Figure 16:**
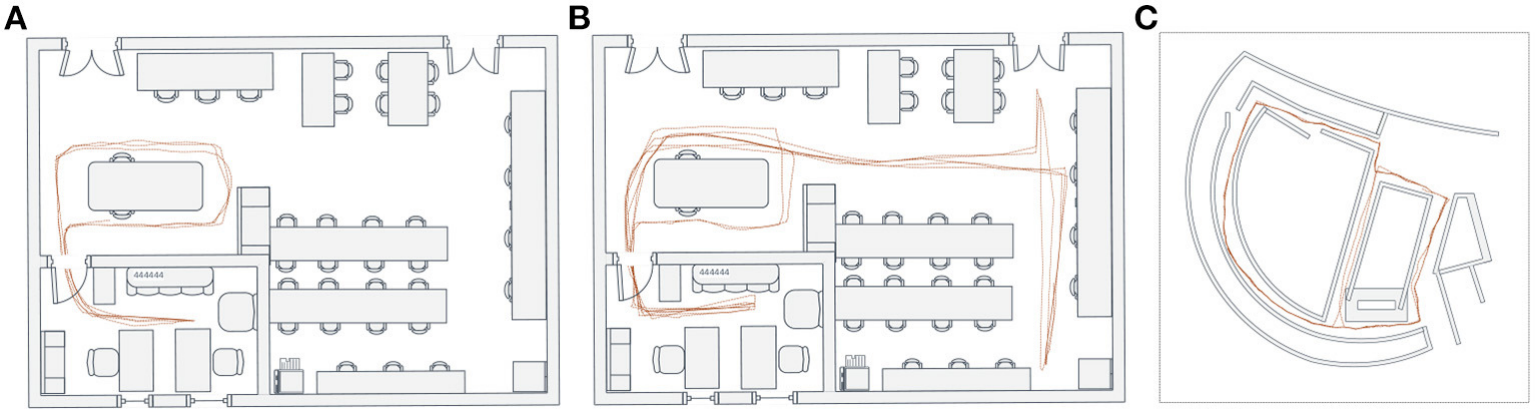
Cognitive map building. **(A,B)** The cognitive map building results based on the *NCRC Lab* dataset. **(C)** The cognitive map building result based on the *Singapore Office* dataset.

As observed from the above experimental results, our system can be successfully driven to produce a cognitive map reflecting the robot's spatial experience and the environmental topological structure (Figure 16C). We also run our system in two other environments and further test its performance of cognitive map building, as shown in Figures 16A,B. The above experimental results, to a large degree, prove that the combination of dead reckoning based on spatial cells and visual cues makes it possible to complete reasonable and effective environmental map building.

### 3.6. GC-RRT^*^ for Path Planning

**Figure 17:**
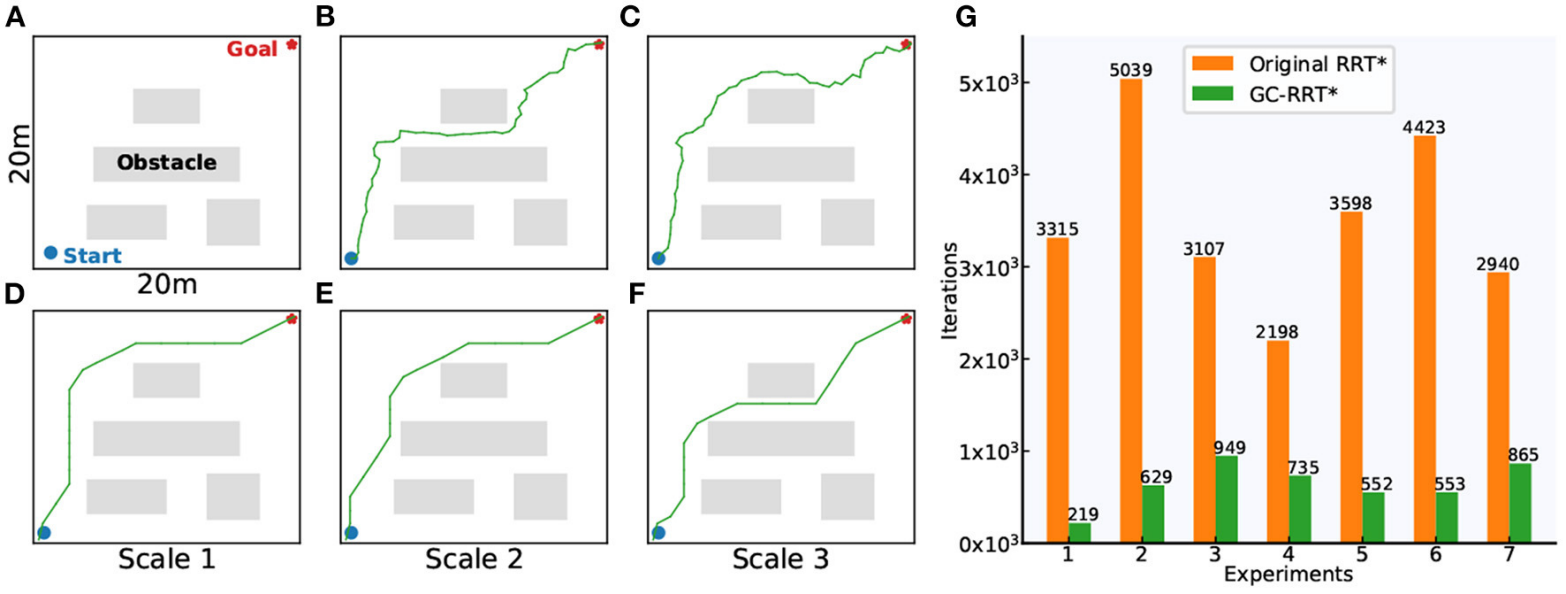
Motion planning experiments. **(A)** The 20 × 20 m experiment environment with spatial resolution of 0.1 m. **(B,C)** The planning results of the original RRT*. **(D–F)** The planning results of GC-RRT*. **(G)** The statistic results of the number of iterations for path planning in the original RRT* and GC-RRT*.

The grid-cell-based multi-scale path planning is shown by the agent's path planning from the start position to the goal position in a 20 × 20 m square environment with several obstacles, as shown in Figure 17A. Path planning using the original RRT^*^ and GC-RRT^*^, respectively, are carried out with the same spatial resolution of 0.1 m.

Figures 17B,C show two path-planning results of the original RRT^*^. Figures 17D–F show the multi-scale path planning results of GC-RRT^*^. It can be seen from the experimental results of 7 trails in Figure 17G that the distributed grid-like firing patterns of grid cells can not only provide an internal multi-scale path planning mechanism but can also achieve a greater chance of success for the agent's path planning.

## 4. Discussion

Encoding spatial information through a path integration mechanism in grid cells may provide an efficient way for a robot to learn the topological structure of the environment. This paper presents a cognitive map building system and shows how the robot can build a topological map of the environment by integrating neuronal activities and vision-assisted map correction. To be workable in real environments, optimized mechanisms for path integration and novel hierarchical vision processing are proposed, helping to achieve the successful transition from a computational model to workable mobile robot application. Burak's model is undoubtedly a representative single-scale grid cell model for path integration. When used for multi-scale extension of grid cells, however, it involves more parameters, and tuning the corresponding parameter representing grid periods fails to obtain grid firing patterns with specific scales we want. In order to enhance the model's usability, we present an optimized grid- cell-modeling mechanism for path integration in order to make the multi-scale extension easy to use. To meet lightweight and real-time requirements, most cognitive map models tend to follow simple vision processing, in which visual template matching is done for loop closure detection. And in these models, visual templates are generally organized into a linear sequence and the matching (between images representing the current visual scene and visual templates) time linearly increases as visual templates accumulate, making the matching process quite time-consuming. In our work, a novel visual template organization method based on a hierarchical structure is proposed to speed up the loop closure detection process. The result is that the matching time fluctuates in a certain range, but does not linearly increase. Besides, neuroscience researches demonstrate that place cells receive information from different sensory sources and visual sensory inputs can also supply important contextual information for the formation and recollection of place field (Jeffery, 2007; Chen et al., 2013; Geva-Sagiv et al., 2015). To achieve a more biologically plausible model, we will include the influence of visual sensory input on place cell tuning into our system in the next work.

This work triggers our rethinking about the relationship between neuroscience and computing science. Computing science provides models for simulating neuroscientific phenomenons (e.g., firing patterns of spatial cells). In turn, further neuroscientific findings provide computing science with guidance on the function and time performance optimization of computational models. In future work, the interaction between neuroscience and computing science will be further and thoroughly explored and fully used to push spatial cognition in a more real-time and intelligent direction.

## Data Availability Statement

The original contributions presented in the study are included in the article/supplementary materials, further inquiries can be directed to the corresponding author/s.

## Author Contributions

All authors provided contributions to study conception and design, acquisition of data or analysis and interpretation of data, drafting the article or revising it critically for important intellectual content, and final approval of the version to be published.

## Conflict of Interest

The authors declare that the research was conducted in the absence of any commercial or financial relationships that could be construed as a potential conflict of interest.
